# An improved error bound for linear complementarity problems for *B*-matrices

**DOI:** 10.1186/s13660-017-1414-z

**Published:** 2017-06-20

**Authors:** Lei Gao, Chaoqian Li

**Affiliations:** 1 School of Mathematics and Information Science, Baoji University of Arts and Sciences, Baoji, Shannxi 721013 P.R. China; 2 School of Mathematics and Statistics, Yunnan University, Kunming, Yunnan 650091 P.R. China

**Keywords:** 90C33, 60G50, 65F35, error bound, linear complementarity problem, *B*-matrix

## Abstract

A new error bound for the linear complementarity problem when the matrix involved is a *B*-matrix is presented, which improves the corresponding result in (Li *et al.* in Electron. J. Linear Algebra 31(1):476-484, 2016). In addition some sufficient conditions such that the new bound is sharper than that in (García-Esnaola and Peña in Appl. Math. Lett. 22(7):1071-1075, 2009) are provided.

## Introduction

Given an $n\times n$ real matrix *M* and $q\in R^{n}$, the linear complementarity problem (LCP) is to find a vector $x\in R^{n}$ satisfying

1 $$ x\geqslant0,\qquad Mx+q\geqslant0, \qquad(Mx+q)^{T}x=0 $$

or to show that no such vector *x* exists. We denote this problem (1) by $\operatorname{LCP}(M, q)$. The $\operatorname{LCP}(M, q)$ arises in many applications such as finding Nash equilibrium point of a bimatrix game, the network equilibrium problem, the contact problem and the free boundary problem for journal bearing etc.; for details, see [3–5].

It is well known that the $\operatorname{LCP}(M, q)$ has a unique solution for any vector $q\in R^{n}$ if and only if *M* is a *P*-matrix [4]. Here a matrix *M* is called a *P*-matrix if all its principal minors are positive. For the $\operatorname{LCP}(M, q)$, one of the interesting problems is to estimate

2 $$ \max_{d\in[0,1]^{n}}\big\| (I -D+DM)^{-1}\big\| _{\infty}, $$

which can be used to bound the error $\|x-x^{*}\|_{\infty}$ [6], that is,

$$\big\| x-x^{*}\big\| _{\infty} \leqslant\max_{d\in[0,1]^{n}}\big\| (I -D+DM)^{-1}\big\| _{\infty} \big\| r(x)\big\| _{\infty}, $$

where $x^{*}$ is the solution of the $\operatorname{LCP}(M, q)$, $r(x)=\min\{ x,Mx+q\}$, $D=\operatorname{diag}(d_{i})$ with $0\leqslant d_{i} \leqslant1$ for each $i\in N$, $d=[d_{1},d_{2},\ldots,d_{n}]^{T}\in[0,1]^{n}$, and the min operator $r(x)$ denotes the componentwise minimum of two vectors.

When the matrix *M* for the $\operatorname{LCP}(M, q)$ belongs to *P*-matrices or some subclass of *P*-matrices, various bounds for (2) were proposed; *e.g.*, see [2, 6–15] and the references therein. Recently, García-Esnaola and Peña in [2] provided an upper bound for (2) when *M* is a *B*-matrix as a subclass of *P*-matrices. Here, a matrix $M=[m_{ij}]\in R^{n, n}$ is called a *B*-matrix [16] if for each $i\in N=\{1,2,\ldots,n\}$,

$$\sum_{k\in N}m_{ik}>0, \quad\text{and}\quad \frac{1}{n} \biggl(\sum_{k\in N}m_{ik} \biggr)>m_{ij}\quad \text{for any }j\in N\text{ and }j\neq i. $$

### Theorem 1

[2], Theorem 2.2

*Let* $M=[m_{ij}]\in R^{n, n}$ *be a* *B*-*matrix with the form*

3 $$ M=B^{+}+C, $$

*where*

4 $$ B^{+} =[b_{ij}]= \left [ \textstyle\begin{array}{c@{\quad}c@{\quad}c} m_{11}-r_{1}^{+} &\cdots &m_{1n}-r_{1}^{+} \\ \vdots & &\vdots \\ m_{n1}-r_{n}^{+} &\cdots &m_{nn}-r_{n}^{+} \end{array}\displaystyle \right ],\qquad C=\left [ \textstyle\begin{array}{c@{\quad}c@{\quad}c} r_{1}^{+} &\cdots &r_{1}^{+} \\ \vdots & &\vdots \\ r_{n}^{+} &\cdots &r_{n}^{+} \end{array}\displaystyle \right ], $$

*and* $r_{i}^{+}=\max\{ 0,m_{ij}|j\neq i\}$. *Then*

5 $$ \max_{d\in [0,1]^{n}}\big\| (I-D+DM)^{-1}\big\| _{\infty} \leqslant\frac{n-1}{\min\{\beta,1\}}, $$

*where* $\beta=\min_{i\in N}\{\beta_{i}\}$ *and* $\beta_{i}=b_{ii}-\sum_{j\neq i}|b_{ij}|$.

It is not difficult to see that the bound (5) will be inaccurate when the matrix *M* has very small value of $\min_{i\in N}\{b_{ii}-\sum_{j\neq i}|b_{ij}|\}$; for details, see [17, 18]. To conquer this problem, Li *et al.*, in [1] gave the following bound for (2) when *M* is a *B*-matrix, which improves those provided by Li and Li in [17, 18].

### Theorem 2

[1], Theorem 2.4

*Let* $M=[m_{ij}]\in R^{n, n}$ *be a* *B*-*matrix with the form* $M=B^{+}+C$, *where* $B^{+}=[b_{ij}]$ *is the matrix of* (4). *Then*

6 $$ \max_{d\in [0,1]^{n}}\big\| (I-D+DM)^{-1}\big\| _{\infty} \leqslant \sum_{i=1}^{n}\frac{n-1}{\min\{\bar{\beta}_{i},1\}} \prod_{j=1}^{i-1}\frac{b_{jj}}{\bar{\beta}_{j}}, $$

*where* $\bar{\beta}_{i}=b_{ii}-\sum_{j=i+1}^{n}|b_{ij}|l_{i}(B^{+})$ *with* $l_{k}(B^{+})=\max_{k\leq i\leq n} \{\frac{1}{|b_{ii}|}\sum_{j=k,\atop j\neq i}^{n}|b_{ij}| \}$, *and* $\prod_{j=1}^{i-1}\frac{b_{jj}}{\bar{\beta}_{j}}=1$ *if* $i=1$.

In this paper, we further improve error bounds on the $\operatorname{LCP}(M,q)$ when *M* belongs to *B*-matrices. The rest of this paper is organized as follows: In Section 2 we present a new error bound for (2), and then prove that this bound is better than those in Theorems 1 and 2. In Section 3, some numerical examples are given to illustrate our theoretical results obtained.

## Main result

In this section, an upper bound for (2) is provided when *M* is a *B*-matrix. Firstly, some definitions, notation and lemmas which will be used later are given as follows.

A matrix $A=[a_{ij}]\in C^{n,n}$ is called a strictly diagonally dominant (*SDD*) matrix if $|a_{ii}|>\sum_{j\neq i}^{n}|a_{ij}|$ for all $i=1,2,\ldots,n$. A matrix $A=[a_{ij}]\in R^{n,n}$ is called a nonsingular *M*-matrix if its inverse is nonnegative and all its off-diagonal entries are nonpositive [3]. In [16] it was proved that a *B*-matrix has positive diagonal elements, and a real matrix *A* is a *B*-matrix if and only if it can be written in the form (3) with $B^{+}$ being a *SDD* matrix. Given a matrix $A=[a_{ij}]\in C^{n,n}$, let

7 $$ \begin{gathered} w_{ij}(A)=\frac{|a_{ij}|}{|a_{ii}|-\sum_{k=j+1,\atop k\neq i}^{n}|a_{ik}|},\quad i\neq j, \\ w_{i}(A)=\max_{j\neq i}\bigl\{ w_{ij}(A) \bigr\} , \\ m_{ij}(A)=\frac{|a_{ij}|+\sum_{ k=j+1,\atop k\neq i}^{n}|a_{ik}|w_{k}(A)}{|a_{ii}|},\quad i\neq j. \end{gathered}$$

### Lemma 1

[19], Theorem 14

*Let* $A=[a_{ij}]$ *be an* $n\times n$ *row strictly diagonally dominant* *M*-*matrix*. *Then*

$$\big\| A^{-1}\big\| _{\infty}\leqslant\sum_{i=1}^{n} \Biggl(\frac{1}{a_{ii}-\sum_{k=i+1}^{n}|a_{ik}|m_{ki}(A)}\prod_{j=1}^{i-1} \frac{1}{1-u_{j}(A)l_{j}(A)} \Biggr), $$

*where* $u_{i}(A)=\frac{1}{|a_{ii}|}\sum_{j=i+1}^{n}|a_{ij}|$, $l_{k}(A)=\max_{k\leq i\leq n} \{\frac{1}{|a_{ii}|}\sum_{j=k,\atop j\neq i}^{n}|a_{ij}| \}$, $\prod_{j=1}^{i-1}\frac{1}{1-u_{j}(A)l_{j}(A)}=1$ *if* $i=1$, *and* $m_{ki}(A)$ *is defined as in* (7).

### Lemma 2

[17], Lemma 3

*Let* $\gamma> 0$ *and* $\eta\geqslant0 $. *Then*, *for any* $x\in [0,1]$,

$$\frac{1}{1-x+\gamma x} \leqslant \frac{1}{\min\{\gamma,1\}} $$

*and*

$$\frac{\eta x}{1-x+\gamma x} \leqslant \frac{\eta}{\gamma}. $$

### Lemma 3

[18], Lemma 5

*Let* $A=[a_{ij}]$ *with* $a_{ii}>\sum_{j=i+1}^{n}|a_{ij}|$ *for each* $i\in N$. *Then*, *for any* $x_{i}\in[0,1]$,

$$\frac{1-x_{i}+a_{ii}x_{i}}{1-x_{i}+a_{ii}x_{i}-\sum_{j=i+1}^{n}|a_{ij}|x_{i}} \leqslant \frac{a_{ii}}{a_{ii}-\sum_{j=i+1}^{n}|a_{ij}|}. $$

Lemmas 2 and 3 will be used in the proofs of the following lemma and Theorem 3.

### Lemma 4

*Let* $M=[m_{ij}]\in R^{n, n}$ *be a* *B*-*matrix with the form* $M=B^{+}+C$, *where* $B^{+}=[b_{ij}]$ *is the matrix of* (4). *And let* $B_{D}^{+}=I-D+DB^{+}=[\tilde{b}_{ij}]$ *where* $D=\operatorname{diag}(d_{i})$ *with* $0\leqslant d_{i} \leqslant1$. *Then*

$$w_{i}\bigl(B_{D}^{+}\bigr)\leqslant \max _{j\neq i} \biggl\{ \frac{|b_{ij}|}{b_{ii}-\sum_{k=j+1,\atop k\neq i}^{n}|b_{ik}|} \biggr\} $$

*and*

$$m_{ij}\bigl(B_{D}^{+}\bigr)\leqslant v_{ij}\bigl(B^{+}\bigr)< 1, $$

*where* $w_{i}(B_{D}^{+})$, $m_{ij}(B_{D}^{+})$ *are defined as in* (7), *and*

$$v_{ij}\bigl(B^{+}\bigr)=\frac{1}{b_{ii}} \Biggl(|b_{ij}|+\sum_{k=j+1,\atop k\neq i}^{n} \biggl(|b_{ik}|\cdot\max_{h\neq k} \biggl\{ \frac{|b_{kh}|}{b_{kk}-\sum_{l=h+1,\atop l\neq k}^{n}|b_{kl}|} \biggr\} \biggr) \Biggr). $$

### Proof

Note that

$$\bigl[B_{D}^{+}\bigr]_{ij}=\tilde{b}_{ij}= \left \{ \textstyle\begin{array}{l@{\quad}l} 1-d_{i}+d_{i}b_{ij}, &i=j,\\ d_{i}b_{ij}, &i\neq j. \end{array}\displaystyle \right . $$

Since $B^{+}$ is *SDD*, $b_{ii}-\sum_{k=j+1,\atop k\neq i}^{n}|b_{ik}|> |b_{ij}|$ for each $i\neq j$. Hence, by Lemma 2 and (7), it follows that

8 $$ \begin{aligned}[b] w_{i}\bigl(B_{D}^{+}\bigr)&=\max _{j\neq i} \bigl\{ w_{ij}\bigl(B_{D}^{+} \bigr) \bigr\} =\max_{j\neq i} \biggl\{ \frac{|b_{ij}|d_{i}}{1-d_{i}+b_{ii}d_{i}-\sum_{k=j+1,\atop k\neq i}^{n}|b_{ik}|d_{i}} \biggr\} \\ &\leqslant\max_{j\neq i} \biggl\{ \frac{|b_{ij}|}{b_{ii}-\sum_{k=j+1,\atop k\neq i}^{n}|b_{ik}|} \biggr\} < 1. \end{aligned} $$

Furthermore, it follows from (7), (8) and Lemma 2 that for each $i\neq j$ ($j< i\leqslant n$)

$$\begin{aligned} m_{ij}\bigl(B_{D}^{+}\bigr)&=\frac{|b_{ij}|\cdot d_{i}+\sum_{k=j+1,\atop k\neq i}^{n}|b_{ik}|\cdot d_{i}\cdot w_{k}(B_{D}^{+})}{1-d_{i}+b_{ii}\cdot d_{i}} \\ &\leqslant \frac{1}{b_{ii}}\cdot \Biggl(|b_{ij}|+\sum _{k=j+1,\atop k\neq i}^{n}|b_{ik}|\cdot w_{k} \bigl(B_{D}^{+}\bigr) \Biggr) \\ &\leqslant\frac{1}{b_{ii}} \Biggl(|b_{ij}|+\sum _{k=j+1,\atop k\neq i}^{n} \biggl(|b_{ik}|\cdot\max _{h\neq k} \biggl\{ \frac{|b_{kh}|}{b_{kk}-\sum_{l=h+1,\atop l\neq k}^{n}|b_{kl}|} \biggr\} \biggr) \Biggr) \\ &=v_{ij}\bigl(B^{+}\bigr) \\ &< \frac{1}{b_{ii}} \Biggl(|b_{ij}|+\sum _{k=j+1,\atop k\neq i}^{n}|b_{ik}| \Biggr)< 1. \end{aligned}$$

The proof is completed. □

By Lemmas 1, 2, 3 and 4, we give the following bound for (2) when *M* is a *B*-matrix.

### Theorem 3

*Let* $M=[m_{ij}]\in R^{n, n}$ *be a* *B*-*matrix with the form* $M=B^{+}+C$, *where* $B^{+}=[b_{ij}]$ *is the matrix of* (4). *Then*

9 $$ \max_{d\in [0,1]^{n}}\big\| (I-D+DM)^{-1}\big\| _{\infty} \leqslant \sum_{i=1}^{n}\frac{n-1}{\min\{\widehat{\beta}_{i},1\}} \prod_{j=1}^{i-1}\frac{b_{jj}}{\bar{\beta}_{j}}, $$

*where* $\widehat{\beta}_{i}=b_{ii}-\sum_{k=i+1}^{n}|b_{ik}|\cdot v_{ki}(B^{+})$ *with* $v_{ki}(B^{+})$ *is defined in Lemma* 4, $\bar{\beta}_{i}$ *is defined in Theorem* 2, *and* $\prod_{j=1}^{i-1}\frac{b_{jj}}{\bar{\beta}_{j}}=1$ *if* $i=1$.

### Proof

Let $M_{D}=I-D+DM$. Then

$$M_{D}=I-D+DM=I-D+D\bigl(B^{+}+C\bigr)=B_{D}^{+}+C_{D}, $$

where $B_{D}^{+}=I-D+DB^{+}=[\tilde{b}_{ij}]$ and $C_{D}=DC$. Similarly to the proof of Theorem 2.2 in [2], we find that $B_{D}^{+}$ is an *SDD* *M*-matrix with positive diagonal elements and that

10 $$ \big\| M_{D}^{-1}\big\| _{\infty}\leqslant\big\| \bigl(I + \bigl(B^{+}_{D}\bigr)^{-1}C_{D} \bigr)^{-1} \big\| _{\infty}\big\| \bigl(B^{+}_{D} \bigr)^{-1} \big\| _{\infty}\leqslant(n-1) \big\| \bigl(B^{+}_{D} \bigr)^{-1} \big\| _{\infty}. $$

Next, we give an upper bound for $\|(B^{+}_{D} )^{-1} \|_{\infty}$. By Lemma 1, we have

11 $$ \big\| \bigl(B^{+}_{D} \bigr)^{-1} \big\| _{\infty}\leqslant\sum_{i=1}^{n} \Biggl( \frac{1}{1-d_{i}+b_{ii}d_{i}-\sum_{k=i+1}^{n}|b_{ik}|\cdot d_{i}\cdot m_{ki}(B^{+}_{D})}\prod_{j=1}^{i-1} \frac{1}{1-u_{j}(B^{+}_{D})l_{j}(B^{+}_{D})} \Biggr), $$

where

$$u_{j}\bigl(B^{+}_{D}\bigr)=\frac{\sum_{k=j+1}^{n}|b_{jk}|d_{j}}{1-d_{j}+b_{jj}d_{j}},\qquad l_{k}\bigl(B^{+}_{D}\bigr)=\max_{k\leq i\leq n} \biggl\{ \frac{\sum_{j=k,\atop j\neq i}^{n}|b_{ij}|d_{i}}{1-d_{i}+b_{ii}d_{i}} \biggr\} , $$

and

$$m_{ki}\bigl(B^{+}_{D}\bigr)=\frac{|b_{ki}|\cdot d_{k}+\sum_{l=i+1,\atop l\neq k}^{n}|b_{kl}|\cdot d_{k}\cdot w_{l}(B_{D}^{+})}{1-d_{k}+b_{kk}\cdot d_{k}} $$

with $w_{l}(B_{D}^{+})=\max_{h\neq l} \{\frac{|b_{lh}|d_{l}}{1-d_{l}+b_{ll}d_{l}-\sum_{s=h+1,\atop s\neq l}^{n}|b_{ls}|d_{l}} \}$.

By Lemmas 2 and 4, we can easily see that, for each $i\in N$,

12 $$ \begin{aligned}[b]\frac{1}{1-d_{i}+b_{ii}d_{i}-\sum_{k=i+1}^{n}|b_{ik}|\cdot d_{i}\cdot m_{ki}(B^{+}_{D})}&\leqslant \frac{1}{\min \{b_{ii}-\sum_{k=i+1}^{n}|b_{ik}|\cdot m_{ki}(B^{+}_{D}),1 \}} \\ &\leqslant \frac{1}{\min \{b_{ii}-\sum_{k=i+1}^{n}|b_{ik}|\cdot v_{ki}(B^{+}),1 \}} \\ &=\frac{1}{\min \{\widehat{\beta }_{i},1 \}}, \end{aligned}$$

and that, for each $k\in N$,

13 $$ l_{k}\bigl(B^{+}_{D}\bigr)=\max _{k\leq i\leq n} \biggl\{ \frac{\sum_{j=k,\atop j\neq i}^{n}|b_{ij}|d_{i}}{1-d_{i}+b_{ii}d_{i}} \biggr\} \leqslant \max _{k\leq i\leq n} \Biggl\{ \frac{1}{b_{ii}}\sum _{j=k,\atop j\neq i}^{n}|b_{ij}| \Biggr\} =l_{k} \bigl(B^{+}\bigr)< 1. $$

Furthermore, according to Lemma 3 and (13), it follows that, for each $j\in N$,

14 $$ \frac{1}{1-u_{j}(B^{+}_{D})l_{j}(B^{+}_{D})}=\frac {1-d_{j}+b_{jj}d_{j}}{1-d_{j}+b_{jj}d_{j}-\sum_{k=j+1}^{n}|b_{jk}|\cdot d_{j}\cdot l_{j}(B^{+}_{D})}\leqslant\frac{b_{jj}}{\bar{\beta}_{j}}. $$

By (11), (12) and (14), we have

15 $$ \big\| \bigl(B^{+}_{D} \bigr)^{-1} \big\| _{\infty}\leqslant \frac{1}{\min \{\widehat{\beta}_{1},1 \}}+\sum_{i=2}^{n} \Biggl(\frac{1}{\min \{\widehat{\beta}_{i},1 \}}\prod_{j=1}^{i-1} \frac {b_{jj}}{\bar{\beta}_{j}} \Biggr). $$

The conclusion follows from (10) and (15). □

The comparisons of the bounds in Theorems 2 and 3 are established as follows.

### Theorem 4

*Let* $M=[m_{ij}]\in R^{n, n}$ *be a* *B*-*matrix with the form* $M=B^{+}+C$, *where* $B^{+}=[b_{ij}]$ *is the matrix of* (4). *Let* $\bar{\beta}_{i}$ *and* $\widehat{\beta}_{i}$ *be defined in Theorems* 2 *and* 3, *respectively*. *Then*

$$\sum_{i=1}^{n}\frac{n-1}{\min\{\widehat{\beta}_{i},1\} }\prod _{j=1}^{i-1}\frac{b_{jj}}{\bar{\beta}_{j}} \leqslant \sum _{i=1}^{n}\frac{n-1}{\min\{\bar{\beta}_{i},1\}}\prod _{j=1}^{i-1}\frac{b_{jj}}{\bar{\beta}_{j}}. $$

### Proof

Note that

$$\bar{\beta}_{i}=b_{ii}-\sum_{j=i+1}^{n}|b_{ij}|l_{i} \bigl(B^{+}\bigr),\qquad \widehat{\beta }_{i}=b_{ii}- \sum_{k=i+1}^{n}|b_{ik}|v_{ki} \bigl(B^{+}\bigr), $$

and $B^{+}$ is a *SDD* matrix, it follows that for each $i\neq j$ ($j< i\leqslant n$)

$$\begin{aligned} v_{ij}\bigl(B^{+}\bigr)&=\frac{1}{b_{ii}} \Biggl(|b_{ij}|+\sum_{k=j+1,\atop k\neq i}^{n} \biggl(|b_{ik}|\cdot\max_{h\neq k} \biggl\{ \frac{|b_{kh}|}{b_{kk}-\sum_{l=h+1,\atop l\neq k}^{n}|b_{kl}|} \biggr\} \biggr) \Biggr) \\ &< \frac{1}{b_{ii}}\sum_{k=j,\atop k\neq i}^{n}|b_{ik}| \\ &\leqslant \max_{j\leqslant i\leqslant n} \Biggl\{ \frac{1}{b_{ii}}\sum _{k=j,\atop k\neq i}^{n}|b_{ik}| \Biggr\} =l_{j} \bigl(B^{+}\bigr). \end{aligned}$$

Hence, for each $i\in N$

$$\widehat{\beta}_{i}=b_{ii}-\sum _{k=i+1}^{n}|b_{ik}|v_{ki} \bigl(B^{+}\bigr)>b_{ii}-\sum_{k=i+1}^{n}|b_{ik}|l_{i} \bigl(B^{+}\bigr)=\bar{\beta}_{i}, $$

which implies that

$$\frac{1}{\min\{\widehat{\beta}_{i},1\}}\leqslant \frac{1}{\min\{\bar{\beta}_{i},1\}}. $$

This completes the proof. □

Remark here that, when $\bar{\beta}_{i}<1$ for all $i\in N$, then

$$\begin{aligned} \frac{1}{\min\{\widehat{\beta}_{i},1\}}< \frac{1}{\min\{\bar{\beta}_{i},1\}}, \end{aligned}$$

which yields

$$\sum_{i=1}^{n}\frac{n-1}{\min\{\widehat{\beta}_{i},1\} }\prod _{j=1}^{i-1}\frac{b_{jj}}{\bar{\beta}_{j}} < \sum _{i=1}^{n}\frac{n-1}{\min\{\bar{\beta}_{i},1\}}\prod _{j=1}^{i-1}\frac{b_{jj}}{\bar{\beta}_{j}}. $$

Next it is proved that the bound (9) given in Theorem 3 can improve the bound (5) in Theorem 1 (Theorem 2.2 in [2]) in some cases.

### Theorem 5

*Let* $M=[m_{ij}]\in R^{n, n}$ *be a* *B*-*matrix with the form* $M=B^{+}+C$, *where* $B^{+}=[b_{ij}]$ *is the matrix of* (4). *Let* *β*, $\bar{\beta}_{i}$ *and* $\widehat{\beta}_{i}$ *be defined in Theorems* 1, 2 *and* 3, *respectively*, *and let* $\alpha=1+\sum_{i=2}^{n}\prod_{j=1}^{i-1}\frac{b_{jj}}{\bar{\beta}_{j}}$ *and* $\widehat{\beta}=\min_{i\in N}\{\widehat{\beta}_{i}\}$. *If one of the following conditions holds*:

(i) $\widehat{\beta}>1$ *and* $\alpha<\frac{1}{\beta}$;
(ii) $\widehat{\beta}<1$ *and* $\alpha\beta<\widehat{\beta}$,

*then*

$$\sum_{i=1}^{n}\frac{n-1}{\min\{\widehat{\beta}_{i},1\}}\prod _{j=1}^{i-1}\frac{b_{jj}}{\bar{\beta}_{j}}< \frac{n-1}{\min\{\beta,1\}}. $$

### Proof

When $\widehat{\beta}>1$ and $\alpha<\frac{1}{\beta}$, we can easily get

$$\sum_{i=1}^{n}\frac{n-1}{\min\{\widehat{\beta}_{i},1\}}\prod _{j=1}^{i-1}\frac{b_{jj}}{\bar{\beta}_{j}}< \frac{n-1}{\min\{\widehat{\beta},1\}}\sum_{i=1}^{n}\prod _{j=1}^{i-1}\frac{b_{jj}}{\bar{\beta}_{j}}= (n-1)\alpha< \frac{n-1}{\beta}\leqslant\frac{n-1}{\min\{\beta,1\}}. $$

Similarly, for $\widehat{\beta}<1$ and $\alpha\beta<\widehat{\beta}$, the conclusion can be proved directly. □

## Numerical examples

Two examples are given to show that the bound in Theorem 3 is sharper than those in Theorems 1 and 2.

### Example 1

Consider the family of *B*-matrices in [17]:

$$M_{k} = \left [ \textstyle\begin{array}{c@{\quad}c@{\quad}c@{\quad}c} 1.5 &0.5 &0.4 &0.5 \\ -0.1 &1.7 &0.7 &0.6 \\ 0.8 &-0.1\frac{k}{k+1} &1.8 &0.7 \\ 0 & 0.7 &0.8 & 1.8 \end{array}\displaystyle \right ], $$

where $k\geqslant1$. Then $M_{k}=B_{k}^{+}+C_{k}$, where

$$B_{k}^{+} = \left [ \textstyle\begin{array}{c@{\quad}c@{\quad}c@{\quad}c} 1 &0 &-0.1 &0 \\ -0.8 &1 &0 &-0.1 \\ 0 &-0.1\frac{k}{k+1}-0.8 &1 &-0.1\\ -0.8 & -0.1 &0 & 1 \end{array}\displaystyle \right ]. $$

By computations, we have $\beta=\frac{1}{10(k+1)}$, $\bar{\beta}_{1}=\bar{\beta}_{2}=\frac {90k+91}{100k+100}$, $\bar{\beta}_{3}=0.99$, $\bar{\beta}_{4}=1$, $\hat{\beta}_{1}=\frac{820k+828}{900k+900}$, $\hat{\beta}_{2}=0.99$, $\hat{\beta}_{3}=1$ and $\hat{\beta}_{4}=1$. Then it is easy to verify that $M_{k}$ satisfies the condition (ii) of Theorem 5. Hence, by Theorem 1 (Theorem 2.2 in [2]), we have

$$\max_{d\in [0,1]^{4}}\big\| (I-D+DM_{k})^{-1}\big\| _{\infty} \leqslant \frac{4-1}{\min\{\beta,1\}}=30(k+1). $$

It is obvious that

$$30(k+1)\longrightarrow+\infty,\quad \text{when }k\longrightarrow+\infty. $$

By Theorem 2, we find that, for any $k\geqslant1$,

$$\begin{gathered} \max_{d\in [0,1]^{4}}\big\| (I-D+DM_{k})^{-1}\big\| _{\infty} \\ \quad\leqslant 3 \biggl(\frac{1}{\bar{\beta}_{1}}+\frac{1}{\bar{\beta}_{2}}\cdot \frac{1}{\bar {\beta}_{1}}+\frac{1}{\bar{\beta}_{3}}\cdot\frac{1}{\bar{\beta}_{1}\bar{\beta}_{2}} +\frac{1}{\bar{\beta}_{1}\bar{\beta}_{2}\bar{\beta}_{3}} \biggr) \\ \quad=3 \biggl(\frac{100k+100}{90k+91}+\frac{(100k+100)^{2}}{(90k+91)^{2}} +\frac{2(100k+100)^{2}}{0.99(90k+91)^{2}} \biggr)< 14.5193. \end{gathered}$$

By Theorem 3, we find that, for any $k\geqslant1$,

$$\begin{gathered} \max_{d\in [0,1]^{4}}\big\| (I-D+DM_{k})^{-1}\big\| _{\infty} \\ \quad\leqslant 3 \biggl(\frac{1}{\hat{\beta}_{1}}+\frac{1}{\hat{\beta}_{2}}\cdot \frac{1}{\bar {\beta}_{1}}+\frac{1}{\bar{\beta}_{1}\bar{\beta}_{2}} +\frac{1}{\bar{\beta}_{1}\bar{\beta}_{2}\bar{\beta}_{3}} \biggr) \\ \quad=3 \biggl(\frac{900k+900}{820k+828}+\frac{(100k+100)}{0.99(90k+91)} +\frac{1.99(100k+100)^{2}}{0.99(90k+91)^{2}} \biggr) \\ \quad< 3 \biggl(\frac {100k+100}{90k+91}+\frac{(100k+100)^{2}}{(90k+91)^{2}} +\frac{2(100k+100)^{2}}{0.99(90k+91)^{2}} \biggr). \end{gathered}$$

In particular, when $k=1$,

$$\begin{gathered} 3 \biggl(\frac{900k+900}{820k+828}+\frac{(100k+100)}{0.99(90k+91)} +\frac{1.99(100k+100)^{2}}{0.99(90k+91)^{2}} \biggr) \approx13.9878, \\3 \biggl(\frac{100k+100}{90k+91}+\frac{(100k+100)^{2}}{(90k+91)^{2}} +\frac{2(100k+100)^{2}}{0.99(90k+91)^{2}} \biggr) \approx14.3775, \end{gathered}$$

and the bound (5) in Theorem 1 is

$$\frac{4-1}{\min\{\beta,1\}}=30(k+1)=60. $$

When $k=2$,

$$\begin{gathered} 3 \biggl(\frac{900k+900}{820k+828}+\frac{(100k+100)}{0.99(90k+91)} +\frac{1.99(100k+100)^{2}}{0.99(90k+91)^{2}} \biggr) \approx14.0265, \\3 \biggl(\frac{100k+100}{90k+91}+\frac{(100k+100)^{2}}{(90k+91)^{2}} +\frac{2(100k+100)^{2}}{0.99(90k+91)^{2}} \biggr) \approx14.4246, \end{gathered}$$

and the bound (5) in Theorem 1 is

$$\frac{4-1}{\min\{\beta,1\}}=30(k+1)=90. $$

### Example 2

Consider the following family of *B*-matrices:

$$M_{k} = \left [ \textstyle\begin{array}{c@{\quad}c} \frac{1}{k} &\frac{-a}{k} \\ 0 &\frac{1}{k} \end{array}\displaystyle \right ], $$

where $\frac{\sqrt{5}-1}{2}< a<1$ and $\frac{2-a^{2}}{1+a}< k<1$. Then $M_{k}=B_{k}^{+}+C$ with *C* is the null matrix.

By simple computations, we can get

$$\beta=\frac{1-a}{k},\qquad\bar{\beta}_{1}=\frac{1-a^{2}}{k},\qquad\bar{ \beta}_{2}=\frac {1}{k},\qquad\hat{\beta}_{1}= \frac{1}{k}\quad \text{and} \quad\hat{\beta}_{2}=\frac{1}{k}. $$

It is not difficult to verify that $M_{k}$ satisfies the condition (i) of Theorem 5. Thus, the bound (6) of Theorem 2 (Theorem 2.4 in [1]) is

$$\sum_{i=1}^{2}\frac{2-1}{\min\{\bar{\beta}_{i},1\}}\prod _{j=1}^{i-1}\frac{b_{jj}}{\bar{\beta}_{j}}= \frac{k+1}{1-a^{2}}, $$

which is larger than the bound

$$\frac{1}{\min\{\beta,1\}}=\frac{k}{1-a} $$

given by (5) in Theorem 1 (Theorem 2.2 in [2]). However, by Theorem 3 we can get

$$\max_{d\in[0,1]^{2}}\big\| (I-D+DM_{k})^{-1}\big\| _{\infty} \leqslant \frac{2-a^{2}}{1-a^{2}}, $$

which is smaller than the bound (5) in Theorem 1, *i.e.*,

$$\frac{2-a^{2}}{1-a^{2}}< \frac{k}{1-a}. $$

In particular, when $a=\frac{4}{5}$ and $k=\frac{8}{9}$, the bounds in Theorems 1 and 2 are, respectively,

$$\frac{1}{\min\{\beta,1\}}=\frac{k}{1-a}=\frac{360}{81} $$

and

$$\sum_{i=1}^{2}\frac{2-1}{\min\{\bar{\beta}_{i},1\}}\prod _{j=1}^{i-1}\frac{b_{jj}}{\bar{\beta}_{j}}= \frac{k+1}{1-a^{2}}=\frac{425}{81}, $$

while the bound (9) in Theorem 3 is

$$\sum_{i=1}^{2}\frac{2-1}{\min\{\hat{\beta}_{i},1\}}\prod _{j=1}^{i-1}\frac{b_{jj}}{\bar{\beta}_{j}}= \frac{2-a^{2}}{1-a^{2}}=\frac{306}{81}. $$

These two examples show that the bound in Theorem 3 is sharper than those in Theorems 1 and 2.

## Conclusions

In this paper, we give a new error bound for the linear complementarity problem when the matrix involved is a *B*-matrix, which improves those bounds obtained in [2] and [1]. Numerical examples are given to illustrate the corresponding results.
